# Food as a trigger for abdominal angioedema attacks in patients with hereditary angioedema

**DOI:** 10.1186/s13023-018-0832-4

**Published:** 2018-06-05

**Authors:** Urs C. Steiner, Lea Kölliker, Christina Weber-Chrysochoou, Peter Schmid-Grendelmeier, Elsbeth Probst, Walter A. Wuillemin, Arthur Helbling

**Affiliations:** 1 Department of Clinical Immunology, University Hospital Zurich, Zurich, Switzerland; 2 Allergy Unit, Department of Dermatology, University Hospital Zurich, Zurich, Switzerland; 3 Division of Haematology and Central Haematology Laboratory, Department of Internal Medicine, Cantonal Hospital Lucerne and University of Berne, Lucerne, Switzerland; 4 Division of Allergology, Department of Rheumatology,Immunology and Allergology, University Hospital Berne, Berne, Switzerland

**Keywords:** Hereditary angioedema (HAE), Trigger factors, Allergy, Intolerance

## Abstract

**Background:**

Hereditary angioedema with C1 inhibitor deficiency (C1-INH-HAE) is a rare inherited disease. In most HAE-affected subjects, defined trigger factors precede angioedema attacks. Mechanisms of how trigger factors stimulate the contact activation pathway with bradykinin generation are not well elucidated. In recent studies, hypersensitivity reactions and food were stated as relevant triggers. We investigated HAE affected people for possible hypersensitivity reactions or intolerances and their relation in triggering angioedema attacks.

**Methods:**

A questionnaire was filled in, recording date of birth, gender, and self-reported angioedema attacks associated with the ingestion of foodstuffs, administration of drugs, hymenoptera stings and hypersensitivity reactions against inhalation allergens. All participants performed a skin prick test against inhalation allergens and food. In patients who stated an association of possible hypersensitivity with angioedema, a serological ImmunoCAP test was also performed.

**Results:**

From the 27 women and 15 men analyzed, 79% stated trigger factors. From those food was mentioned in 36%. The suspected food included tomato, green salad, fish, citrus fruits, apple, onion, garlic, cheese, chili, kiwi, milk, tree nuts, strawberry, pineapple, shrimps, bread, banana, leek, chicken and alcohol, and were associated with abdominal angioedema. Neither the skin prick test nor the ImmunoCAP-test turned out positive for the tested food allergens.

**Conclusion:**

Food seems to be a relevant trigger factor, causing angioedema in HAE affected patients. The reason, however, is not IgE-mediated hypersensitivity, but most probably an intolerance reaction to food products.

## Background

Hereditary angioedema with C1 inhibitor deficiency (C1-INH-HAE) is a rare inherited disease, due to mutations of the *SERPING1* gene. Two different variants have been described. Type I is characterized by a quantitative decrease in C1-INH protein, in Type II, the C1-INH protein has a normal level, but is dysfunctional [1, 2]. C1-INH inhibits factors of the contact system including FXII and kallikrein, and controls the generation of vasoactive peptides such as bradykinin [3]. C1-INH-HAE is characterized by episodic attacks of subcutaneous and submucosal swelling affecting extremities, trunk, genitals, and face, and might be life threatening when upper airways and abdomen are affected [4, 5]. Although it is a systemic activation process, frequency and localization of angioedema are highly variable, depending on the trigger factors and the upregulation of bradykinin receptors 1 and 2 (B1R, B2R) on the endothelium [6, 7]. In 56 to 91% of HAE-affected subjects, defined factors or circumstances may trigger angioedema attacks [8, 9]. The most frequent trigger factors are emotions, mechanical trauma, infections, and, in women, hormonal causes, such as menstruation, pregnancy or intake of estrogen pills. In recent studies food ingestion has also been suggested to cause angioedema attacks in HAE [8–10]. Mechanisms of how food could trigger angioedema attacks have not yet been elucidated. Patients who are affected by angioedema induced by food, drugs or hymenoptera stings interpret the cause most often as an IgE-mediated hypersensitivity reaction. Recent studies have demonstrated an interaction of activated mast cells by an allergen and the contact activation pathway resulting in the production of bradykinin [11–13].

The aim of this study was to evaluate the influence of atopy or IgE-mediated hypersensitivity reactions to specific food, drugs or hymenoptera stings on angioedema attacks in HAE affected subjects. We assessed the prevalence of atopy in 42 HAE affected patients in correlation with the frequency of angioedema attacks and investigated trigger factors including food, drugs or hymenoptera venoms for specific IgE antibodies.

## Methods

### Patients

Patients from the Division of Haematology of the Cantonal Hospital Lucerne and from the Allergy Units of the University Hospitals of Berne and Zurich were included. They all fulfilled the diagnostic criteria for HAE [1]. All patients included are part of the Swiss HAE cohort study [9].

A questionnaire was sent to all participants: patients’ personal data, details of hypersensitivities/intolerances and symptoms (e.g.rhinoconjunctivitis, asthma, vomiting, stomach pain, diarrhea) were recorded. All participating patients gave their written informed consent. The protocol of this study received Ethics Committee approval from the Northwest - and Central Switzerland ethics committee. The study was conducted according to the principles of good clinical practice and adhered strictly to the ethical standards outlined in the Declaration of Helsinki [14].

### Skin tests

Skin prick tests (SPT) with 7 commercially available aeroallergens (birch, ash, grass, mugwort, *Alternaria alternata*, house dust mite and cat dander) and 13 food antigen extracts (soy, hen’s egg, cow’s milk, celery, apple, codfish, shrimp, peanut, walnut, curry, chili, rye flour, and wheat flour) were tested (ALK-Abello, Hørsholm, Denmark). For banana and pine apple a prick-to-prick test was performed. If the wheal diameter was ≥3 mm the test reaction was considered to be positive as recommended in the European standards for skin prick testing [15].

For the SPT with medications the drug powder dissolved in 0.9% saline was applied onto the skin of the forearms. If a wheel of at least 5 mm in diameter with surrounding erythema after 20 min was evident, the test was found positive [16].

### Serologic tests

Serum samples were tested with the multitest Phadiatop Sx1 (timothy grass, birch, cultivated rye, mugwort, dog dander, cat dander, *Cladosporium herbarum*, *D. pteronyssinus*) and the multitest Fx5 (egg white, cow’s milk, fish (cod), wheat flour, peanut, soy bean). Specific IgE (sIgE) and total IgE were determined by ImmunoCAP FEIA (Thermo Fisher Scientific/Phadia, Uppsala, Sweden) according to the manufacturer’s directions for use. SIgE levels > 0.35 kUA/L (RAST class ≥1) were considered positive. In one patient IgA and anti-transglutaminase IgA was performed (Thermo Fisher Scientific/Phadia, Uppsala, Sweden).

### Data handling

Clinical information was collected in each study center and transmitted to the administration site of the responsible researcher. Data were entered into the electronic database. If data were incomplete, the participant was contacted for specific clarification. The final dataset was pseudonymised and prepared for analysis. The pseudonymisation key is stored at the administration site.

For evaluating attack frequency we considered the results from the Swiss HAE cohort study [9] and assumed that sensitization patterns and attack frequency at the time point of the 2012-evaluation were stable.

## Results

The analysis was based on 42 consenting patients (27 women and 15 men) with a mean age of 45 years (SD 16.1), all completed the questionnaire, conducted a skin test and consented to provide blood samples.

### Trigger factors

Trigger factors causing HAE were indicated by 79% (23 women, 10 men). The most frequent trigger was emotion with 79.% (18 women, 8 men) followed by trauma with 55% (12 women, 6 men) food 36% (10 women, 2 men), drugs 6% and hymenoptera stings 6% with 2 women each. Patient characteristics and trigger factors are presented in Table 1.

**Table 1:** Characteristics of 42 patients

Parameter	Number (men/women)
Patients	42 (15/27)
mean age (years)	45 (SD 16.1)
HAE variants	
Type I	41 (0/41)
Type II	1 (1/0)
Trigger factors	
Emotions	26 (8/18)
Trauma / Mechanical trigger	18 (6/12)
Hormones^a^	17 (0/17)
Infection	13 (3/10)
Food	12 (2/10)
Drugs	2 (0/2)
Bee/Wasp sting	2 (0/2)
Total	33 (10/23)
Atopy	
Patients	12 (3/9)
Grass pollen	10 (1/9)
Tree pollen	10 (3/7)
House dust mite	5 (2/3)
Cat dander	3 (1/2)
Mugwort	3 (1/2)
Self reported hypersensitivities/intolerances inducing angioedema	
Atopic	4 (0/4)
Non atopic	9 (2/7)

^a^menstruation, pregnancy or intake of estrogen pills

### Atopy in HAE affected patients

Atopy documented by positive skin prick tests was identified in 29% (9 women, 3 men). Grass and tree pollen were the most prevalent allergen sources, followed by house dust mites, cat dander and mugwort. Ten of the 12 sensitized subjects were symptomatic with rhinoconjunctivitis (Table 1).

### Association of self-reported hypersensitivity- or intolerance reaction and angioedema

Trigger factors were interpreted by the subjects themselves in 57% (11 women, 2 men), as an IgE-mediated hypersensitivity reaction. Four of these 13 subjects were indeed confirmed to be atopic (Tables 1 and 2). In all the twelve subjects with food as a trigger, abdominal angioedema was induced. The included food were tomato (1), green salad (1), fish (2), citrus fruits (2), apple (3), onion (3), garlic (3), cheese (2), chili (2), kiwi (1), milk (3), tree nut (1), peanut (1), strawberry (2), pineapple (3), shrimps (1), bread (1), banana (1), leek (1), chicken (1), chamomile (1) and alcohol (1). However skin prick test for the specific food was negative in all 12 participants and none of the 11/12 tested patients with multitest fx5 and single food proteins had specific IgE (Table 2). Two women each mentioned bee/wasp venom, and drugs as a trigger. No specific IgE to the recombinant venom allergens of Api m1, Api m10 or Vesv5 were detected. In the two subjects with assumed drug induced angioedema, SPT of acetylsalicylic acid (ASS) in one and ASS combinations Pretuval®, NeoCitran® and the antidepressant Deroxat® (paroxetin) in the other patient resulted negative. Drug intake was not concomitant with reactions to hymenoptera venoms (Table 2). Four subjects complained of urticarial episodes. One atopic patient suffered from acute urticaria, 3 non atopic patients suffered from acute, inducible (physical) and aspirin induced urticaria (AIU) each. The one with AIU mentioned an association with urticaria and HAE typical angioedema. The other three did not observe an association between their urticaria and angioedema.

**Table 2:** Self reported hypersensitivity- and intolerance reactions associated with angioedema (AE) in atopic and non atopic subjects

Patients	Self reported hypersensitivity/intolerance				SPT		Phadiatop Sx1, fx5; sIgE; (CAP class 1–6)			Localisation of AE	AE induced by
	Inhalation allergens	Food	Drug	Bee−/wasp-stings	Aeroallergns^a^	Food^b^/drug	Aeroallergens	Food allergens	Bee−/wasp allergens		
Atopic, associated with AE (*n* = 4)											
Female	trees, grass, hdm, cat	tomato, green salad			positive for: grass, ash, cat	all negative	positive for: grass (4), birch (1), rye (3), cat dander (2)	negative for fx5, tomato,	nd	abdomen	food
Female	trees, grass	fish, citrus fruits			positive for: grass, birch	all negative	positive for: grass (1), rye (1)	negative for: fx5	nd	abdomen	food
Female	hdm	apple, onion, garlic, cheese, chili		bee- and wasp sting	positive for: hdm	all negative	positive for: hdm (1)	negative for: fx5, onion, apple, chili	negative for: rApim1/10, rVes v5	abdomen	food Bee−/wasp sting
Female	trees, grass, hdm, cat	kiwi,onion, chili, fondue		bee and wasp sting	positive for: birch, ash, grass, hdm	all negative	positive for: grass (3), hdm (3)	negative for: fx 5, onion, kiwi, chili	negative for: rApim1/10, rVes v5	abdomen	food Bee−/wasp sting
Non atopic associated with AE (*n* = 9)											
Female	yes, ns	strawberry, chamomile, pineapple			negative	negative	Sx1 negative	negative for: fx5, chamomile, strawberry, pineapple	nd	abdomen	food
Female		garlic, shrimps, milk			negative	negative	Sx1 negative	negative for: fx5, shrimp, garlic	nd	abdomen	food
Female	grass	milk, bread			negative	negative	Sx1 negative	negative for: fx5, anti tg-IgA	nd	abdomen	food
Female		cheese, apple, banana, garlic, onion, leek			negative	negative	nd	nd	nd	abdomen	food
Female	grass, trees, hdm	walnut, pineapple, chicken	ASS		negative	negative for food allergens, ASS	Sx1 negative	negative for: fx5, chicken, pine apple, walnut	nd	ASS: mouth, eyelid food: abdomen	drugs food
Female	yes, ns	pineapple, peanut, apple, orange, strawberry			negative	negative	Sx1 negative	negative for: fx 5, apple, strawberry, orange, pine apple	nd	abdomen face extremities	food
Male		alcohol, milk			negative	negative	Sx1 negative	fx 5 negative	nd	alcohol: extremities, genitals, abdomen milk: abdomen	food
Male		fish			negative	negative	Sx1 negative	fx5 negative	nd	abdomen	food
Female			Paroxetin®, Pretuval®, Neo-citran®		negative	negative for Paroxetin®, Pretuval®, Neo-citran®	Sx1 negative	nd	nd	face, abdomen	drugs

*AE* angioedema, *SPT* skin prick test, *hdm* house dust mites, *ASS* acetylsalicylic acid, *ns* not specified, *nd* not done

^a^SPT: 7 aeroallergens: birch, ash, grass, mugwort, *Alternaria alternata*, house dust mite and cat dander

^b^SPT 14 food allergen extracts: soy, hen’s egg, cow’s milk, celery, apple, codfish, shrimp, peanut, walnut, pepper, curry, rye flour, and wheat flour (aeroallergen extract and food allergen extract by ALK-Abello, Hørsholm, Denmark). Only positive results mentioned

Multitest Sx1: Specific IgE for: timothy grass, birch, cultivated rye, mugwort, dog dander, cat dander, *Cladosporium herbarum*, *D. pteronyssinus*

Multitest fx5: Specific IgE for: egg white, cow’s milk, fish (cod), wheat flour, peanut, soy bean

( ): CAP class 1–4 of allergen specific IgE in kU/l: 0: < 0.35; 1: 0.35–0.7; 2: 0.71–3.5; 3: 3.51–17.5; 4: 17.5–50

Anti tg IgA-Ab: anti transglutaminase IgA antibodies (marker for celiac disease)

### Frequency of angioedema in atopic and non-atopic subjects

There was no difference in the incidence of HAE attacks in atopic and non-atopic subjects (Table 3).

**Table 3:** Frequency and distribution of attacks per year stratified for male vs female and atopy vs non-atopy

	Female (*n* = 27)		* *p*-value	Male (*n* = 15)	
	No atopy (*n* = 18)	Atopy (n = 9)		No atopy (*n* = 12)	Atopy (*n* = 3)
No attacks (# subjects)	2	1		5	3
Rare attacks (# subjects)	4	2	0.029	5	-
# attacks (range)	1.5 (1–3)	4 (4–4)		4 (2–6)	-
Moderate attacks (# subjects)	8	5	0.854	2	-
# attacks (range)	25.1 (15–37)	26 (12–36)		12 (12–12)	-
Frequent attacks (# subjects)	4	1		-	-
# attacks (range)	89.5 (53–136)	93		-	-

* t-test: The *p*-values are for the comparison of attack frequency among atopic and non-atopic subjects within one specific category of attacks, where feasible

no attacks: < 1/year; rare attacks: < 1/month; ≥ 1/year; moderate attacks: < 1/week; ≥ 1/month; frequent attacks: ≥1/week

## Discussion

We investigated 42 participants (31%) of the Swiss HAE cohort for suspected self-reported hypersensitivity or intolerance reactions as trigger factors for their angioedema attacks [9]. The majority of the participating individuals (79%) stated that trigger factors preceded angioedema attacks. Emotions, followed by trauma, were mentioned most frequently, which is in line with the current literature [8, 9]. Hypersensitivities or intolerances to food, medications or hymenoptera stings were stated by almost half of the patients with trigger factors (Table 1).

IgE-mediated hypersensitivity with mast cell activation can be associated with heparin release and a consequent bradykinin generation [11–13, 17, 18]. Almost one third of our cohort was atopic. This proportion reflects the general population’s prevalence of atopy in Northern Europe and in industrialized countries [19–21]. In our cohort no difference of attack frequency was documented between atopic and non atopic subjects. With regard to the question of whether suspected hypersensitivity or intolerance is associated with angioedema, there was no relevant difference between atopic (33%) compared to non-atopic subjects (30%). Based on our data, atopy does not predispose HAE affected subjects to suffer from more frequent angioedema attacks (Table 3).

Interestingly in all 12 subjects the specific food which was mentioned as a trigger factor seemed to induce abdominal angioedema attacks (Table 2). However, in none of them specific IgE against the indicated food products could be documented, neither by SPT nor by serological tests. Therefore, food induced abdominal angioedema attacks in these subjects are triggered most probably by an intolerance reaction due to unknown mechanisms. Most of the food mentioned by the investigated subjects such as cheese, alcoholic beverages, fish, tomatoes, strawberries, pineapples, nuts, citrus fruits and kiwis contain or release histamine [22]. Therefore a histamine intolerance reaction is probably associated with the induction of angioedema. However, a clear allocation of food inducing histamine intolerance remains difficult and a reliable laboratory test for objective diagnosis is lacking [23–25]. Three patients suffered from a known lactose intolerance and they stated a clear association between HAE attacks and intake of cow’s milk products. In lactose intolerance the unabsorbed lactose leads to osmotic diarrhea and gas production causing flatulence and abdominal pain. In these subjects also a heightened activity of the innate mucosal immune system with increased counts of mast cells and lymphocytes is described [26, 27]. Which of these mechanisms are influencing the formation of angioedema in HAE remains to be analyzed further. For one subject, who declared bread as a cause of angioedema celiac disease was excluded with a negative test for anti-tissue transglutaminase IgA-antibodies.

This study corroborates that food is a relevant trigger factor for abdominal angioedema attacks in HAE affected subjects. However the underlying pathomechanisms for activation of the contact activation pathway with bradykinin formation in the investigated subjects seem to be intolerance reactions rather than IgE-mediated hypersensitivity. In these patients a careful individual nutritional counseling is recommended with the aim of a targeted avoidance of the food concerned.

Little data are available on urticaria in HAE [28, 29]. The prevalence of urticaria in our cohort was 10%. This proportion was lower compared to the general population or a recently described survey of a HAE cohort, which is most probably explained by the small number of our investigated population [28, 30].

An association of urticaria with typical HAE was only corroborated by the patient with AIU. Acute and physical urticaria did not seem to provoke HAE. These findings are emphasizing that mechanisms in AIU are different compared to acute or physical urticaria. In AIU most probably activated mast cells are involved but the exact pathomechanism is still not clear [31]. The drugs ASS, ASS combinations and Paroxetin® provoked peripheral, facial and abdominal angioedema. According to the negative skin test results, non IgE-mediated hypersensitivity reaction (e.g. pseudoallergy) most probably induced the symptoms [32, 33]. Further investigations are required to analyze the threshold of mast cell activation in the different subtypes of urticaria and pseudoallergy with a subsequent potential to induce the contact activation pathway with bradykinin release.

Two patients who stated a local reaction and an abdominal angioedema attack induced by hymenoptera stings, showed no sensitization to the major allergens rApi m1/10 and rVes v5 involved. In these patients, it was not allergy, but rather the pain of the sting and the stress that triggered the angioedema attacks.

Severe IgE-mediated hypersensitivity reactions most probably can induce bradykinergic angioedema attacks by induction of the contact activation pathway [12, 13]. Therefore if HAE affected subjects indicate triggers such as food, hymenoptera stings or drugs, which have the potential to induce severe IgE-mediated hypersensitivity reaction, specific IgE antibodies must be excluded. However intolerance reactions seem to be more important triggers than IgE-mediated mechanisms.

### Strength and limitations

To our knowledge this is the first study that addresses food as a trigger factor in patients with HAE. Although the selected cohort is small, we consider the results valid. Self-reporting of trigger factors and symptoms carry the risk of reporting bias. A downside of this study is that no provocation tests with the incriminated food, suspected drug or a challenge sting test with a living hymenoptera has been performed.

## Conclusion

Food products can be important trigger factors for abdominal angioedema attacks in HAE. The underlying pathomechanism most often is compatible with an intolerance reaction. IgE-mediated hypersensitivity reactions seem rarely cause angioedema in HAE. Studying trigger factors in HAE is essential to obtain a better understanding of the disease mechanisms.

## Abbreviations

AIU: Aspirin induced urticaria
ASS: Acetylsalicylic acid
C1-INH-HAE: Hereditary angioedema with C1 inhibitor deficiency
sIgE: Specific IgE
SPT: Skin prick test

## References

[1] Cicardi M, Aberer W, Banerji A, Bas M, Bernstein JA, Bork K (2014). Classification, diagnosis, and approach to treatment for angioedema: consensus report from the hereditary angioedema international working group. Allergy.

[2] Rosen FS, Pensky J, Donaldson V, Charache P (1965). Hereditary angioneurotic edema: two genetic variants. Science.

[3] Nussberger J, Cugno M, Cicardi M (2002). Bradykinin-mediated angioedema. N Engl J Med.

[4] Lumry WR, Castaldo AJ, Vernon MK, Blaustein MB, Wilson DA, Horn PT (2010). The humanistic burden of hereditary angioedema: impact on health-related quality of life, productivity, and depression. Allergy Asthma Proc.

[5] Bork K, Hardt J, Schicketanz KH, Ressel N (2003). Clinical studies of sudden upper airway obstruction in patients with hereditary angioedema due to C1 esterase inhibitor deficiency. Arch Intern Med.

[6] Agostoni A, Cicardi M (1992). Hereditary and acquired C1-inhibitor deficiency: biological and clinical characteristics in 235 patients. Medicine (Baltimore).

[7] Hofman ZL, Relan A, Zeerleder S, Drouet C, Zuraw B, Hack CE (2016). Angioedema attacks in patients with hereditary angioedema: local manifestations of a systemic activation process. J Allergy Clin Immunol.

[8] Zotter Z, Csuka D, Szabo E, Czaller I, Nebenfuhrer Z, Temesszentandrasi G (2014). The influence of trigger factors on hereditary angioedema due to C1-inhibitor deficiency. Orphanet J Rare Dis.

[9] Steiner UC, Weber-Chrysochoou C, Helbling A, Scherer K, Grendelmeier PS, Wuillemin WA (2016). Hereditary angioedema due to C1 - inhibitor deficiency in Switzerland: clinical characteristics and therapeutic modalities within a cohort study. Orphanet J Rare Dis.

[10] Wais-Nocker B, Steiner U, Spath P, Wuillemin WA (2010). Clinical facets of hereditary angioedema among Swiss patients. Praxis (Bern 1994).

[11] van der Linden PW, Hack CE, Eerenberg AJ, Struyvenberg A, van der Zwan JK (1993). Activation of the contact system in insect-sting anaphylaxis: association with the development of angioedema and shock. Blood.

[12] Oschatz C, Maas C, Lecher B, Jansen T, Bjorkqvist J, Tradler T (2011). Mast cells increase vascular permeability by heparin-initiated bradykinin formation in vivo. Immunity.

[13] Sala-Cunill A, Bjorkqvist J, Senter R, Guilarte M, Cardona V, Labrador M (2015). Plasma contact system activation drives anaphylaxis in severe mast cell-mediated allergic reactions. J Allergy Clin Immunol.

[14] The Helsinki Declaration of the World Medical Association (WMA) (2014). Ethical principles of medical research involving human subjects. Pol Merkur Lekarski.

[15] Heinzerling L, Mari A, Bergmann KC, Bresciani M, Burbach G, Darsow U (2013). The skin prick test - European standards. Clin Transl Allergy.

[16] Brockow K, Garvey LH, Aberer W, Atanaskovic-Markovic M, Barbaud A, Bilo MB (2013). Skin test concentrations for systemically administered drugs -- an ENDA/EAACI drug allergy interest group position paper. Allergy.

[17] Hofman Z, de Maat S, Hack CE, Maas C (2016). Bradykinin: inflammatory product of the coagulation system. Clin Rev Allergy Immunol.

[18] Guilarte M, Sala-Cunill A, Luengo O, Labrador-Horrillo M, Cardona V (2017). The mast cell, contact, and coagulation system connection in anaphylaxis. Front Immunol.

[19] Wuthrich B, Schmid-Grendelmeier P, Schindler C, Imboden M, Bircher A, Zemp E (2013). Prevalence of atopy and respiratory allergic diseases in the elderly SAPALDIA population. Int Arch Allergy Immunol.

[20] Haftenberger M, Laussmann D, Ellert U, Kalcklosch M, Langen U, Schlaud M (2013). Prevalence of sensitisation to aeraoallergens and food allergens: results of the German health interview and examination survey for adults (DEGS1). Bundesgesundheitsblatt Gesundheitsforschung Gesundheitsschutz.

[21] Abramson MJ, Schindler C, Schikowski T, Bircher AJ, Burdet L, Gerbase MW (2016). Rhinitis in Swiss adults is associated with asthma and early life factors, but not second hand tobacco smoke or obesity. Allergol Int.

[22] San Mauro Martin I, Brachero S, Garicano Vilar E (2016). Histamine intolerance and dietary management: a complete review. Allergol Immunopathol (Madr).

[23] Komericki P, Klein G, Reider N, Hawranek T, Strimitzer T, Lang R (2011). Histamine intolerance: lack of reproducibility of single symptoms by oral provocation with histamine: a randomised, double-blind, placebo-controlled cross-over study. Wien Klin Wochenschr.

[24] Reese I (2014). Debating histamine intolerance: are adverse reactions to histamine-containing foods fact or fiction?. Hautarzt.

[25] Reese I, Ballmer-Weber B, Beyer K, Fuchs T, Kleine-Tebbe J, Klimek L (2017). German guideline for the management of adverse reactions to ingested histamine: guideline of the German Society for Allergology and Clinical Immunology (DGAKI), the German Society for Pediatric Allergology and Environmental Medicine (GPA), the German Association of Allergologists (AeDA), and the Swiss Society for Allergology and Immunology (SGAI). Allergo J Int.

[26] Deng Y, Misselwitz B, Dai N, Fox M (2015). Lactose intolerance in adults: biological mechanism and dietary management. Nutrients.

[27] Yang J, Fox M, Cong Y, Chu H, Zheng X, Long Y (2014). Lactose intolerance in irritable bowel syndrome patients with diarrhoea: the roles of anxiety, activation of the innate mucosal immune system and visceral sensitivity. Aliment Pharmacol Ther.

[28] Rasmussen ER, de Freitas PV, Bygum A (2016). Urticaria and prodromal symptoms including erythema Marginatum in Danish patients with hereditary angioedema. Acta Derm Venereol.

[29] Martin L, Renne T, Drouet C (2016). Urticaria as a presenting prodromal manifestation of attacks of hereditary angioedema. Acta Derm Venereol.

[30] Zuberbier T, Aberer W, Asero R, Abdul Latiff AH, Baker D, Ballmer-Weber B, et al. The EAACI/GA(2)LEN/EDF/WAO Guideline for the Definition, Classification, Diagnosis and Management of Urticaria. The 2017 Revision and Update. Allergy. 2018. (Epub ahead of print). 10.1111/all.13397 29336054

[31] Yamashita M (2016). Aspirin intolerance: experimental models for bed-to-bench. Curr Drug Targets.

[32] Schnyder B, Brockow K (2015). Pathogenesis of drug allergy--current concepts and recent insights. Clin Exp Allergy.

[33] Modena B, White AA, Woessner KM (2017). Aspirin and nonsteroidal Antiinflammatory drugs hypersensitivity and management. Immunol Allergy Clin N Am.

